# Improved visualization of the bone-implant interface and osseointegration in *ex vivo* acetabular cup implants using photon-counting detector CT

**DOI:** 10.1186/s41747-023-00335-y

**Published:** 2023-05-01

**Authors:** Mischa Woisetschläger, Ronald Booij, Erik Tesselaar, Edwin H. G. Oei, Jörg Schilcher

**Affiliations:** 1 Department of Radiology and Department of Health, Medicine and Caring Sciences, Linköping University, Linköping, Sweden; 2 Center for Medical Image Science and Visualization (CMIV), Linköping University, Linköping, Sweden; 3 Department of Radiology & Nuclear Medicine, Erasmus Medical Center, Rotterdam, The Netherlands; 4 Department of Medical Radiation Physics, and Department of Health, Medicine and Caring Sciences, Linköping University, Linköping, Sweden; 5 Department of Orthopedics, Department of Clinical and Experimental Medicine, Linköping University, Linköping, Sweden; 6 Wallenberg Center for Molecular Medicine, Linköping University, Linköping, Sweden

**Keywords:** Arthroplasty, Joint prosthesis, Osseointegration, Osteoarthritis (hip), Tomography (x-ray computed)

## Abstract

**Background:**

Successful osseointegration of joint replacement implants is required for long-term implant survival. Accurate assessment of osseointegration could enable clinical discrimination of failed implants from other sources of pain avoiding unnecessary surgeries. Photon-counting detector computed tomography (PCD-CT) provides improvements in image resolution compared to conventional energy-integrating detector CT (EID-CT), possibly allowing better visualization of bone-implant-interfaces and osseointegration. The aim of this study was to assess the quality of visualization of bone-implant-interfaces and osseointegration in acetabular cup implants, using PCD-CT compared with EID-CT.

**Methods:**

Two acetabular implants (one cemented, one uncemented) retrieved during revision surgery were scanned using PCD-CT and EID-CT at equal radiation dose. Images were reconstructed using different reconstruction kernels and iterative strengths. Delineation of the bone-implant and bone-cement-interface as an indicator of osseointegration was scored subjectively for image quality by four radiologists on a Likert scale and assessed quantitatively.

**Results:**

Delineation of bone-implant and bone-cement-interfaces was better with PCD-CT compared with EID-CT (*p* ≤ 0.030). The highest ratings were given for PCD-CT at sharper kernels for the cemented cup (PCD-CT, median 5, interquartile range 4.25–5.00 *versus* EID-CT, 3, 2.00–3.75, *p* < 0.001) and the uncemented cup (5, 4.00–5.00 *versus* 2, 2–2, respectively, *p* < 0.001). The bone-implant-interface was 35–42% sharper and the bone-cement-interface was 28–43% sharper with PCD-CT compared with EID-CT, depending on the reconstruction kernel.

**Conclusions:**

PCD-CT might enable a more accurate assessment of osseointegration of orthopedic joint replacement implants.

**Key points:**

• The bone-implant interface *ex vivo* showed superior visualization using photon-counting detector computed tomography (PCD-CT) compared to energy-integrating detector computed tomography.

• Harder reconstruction kernels in PCD-CT provide sharper images with lower noise levels.

• These improvements in imaging might make it possible to visualize osseointegration *in vivo*.

## Background

**Fig. 1:**
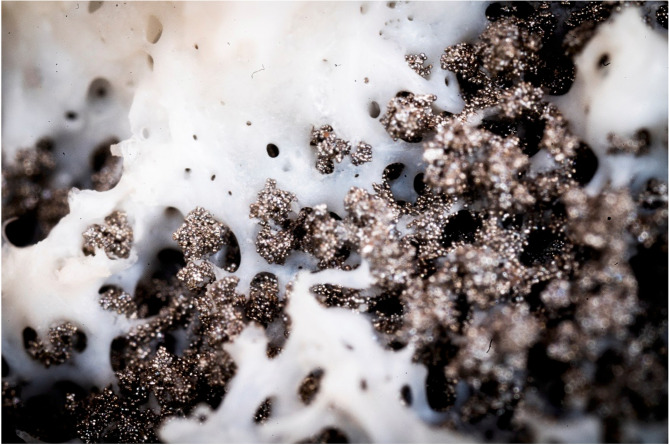
Bone ingrowth into the porous coated surface of an uncemented cup (Trident® Tritanium™, revision, Acetabular System, Stryker Orthopaedics). White (bone), grey (titanium beads). Courtesy of Thor Balkhed, Linköping University

The number of total joint replacements (TJRs) performed annually to treat end-stage osteoarthritis is continuously increasing [1]. Only in the USA, more than 1.5 million first-time TJRs of the hip and knee are estimated to be performed per year in 2030 [2]. However, a TJR has a limited lifetime [3]. Therefore, the number of reoperations performed to treat a failed TJR are also increasing [4]. The main reason for implant revision is aseptic loosening. In other words, the implant loses its direct structural and functional connection with the host bone [5]. This direct connection between the implant and host bone is referred to as osseointegration and involves the formation of a direct bony interdigitation between the implant and the bone (Fig. 1) [6]. The process of osseointegration builds upon the unique capacity of bony tissue to reactivate embryonal processes for healing instead of healing with scar tissue [7]. This type of biological anchorage of an artificial joint bearing implant is the prerequisite for an asymptomatic joint replacement maintained during functional loading over a prolonged period of time [8]. When the implant fails, patients develop symptoms and functional deficits, which can lead to reoperation.

With the growing number of patients with TJR, the ability to discriminate a loosened TJR from other differential diagnoses such as benign musculoskeletal sources of pain (*e.g.*, degenerative changes in the pelvis or spine) has become urgent. However, only histological analyses based on tissue samples acquired during surgery, have been able to assess the state of osseointegration [9]. Neither clinical, radiological nor any other tools have been available to reliably assess osseointegration *in vivo* among other differential diagnoses in patients after total joint replacement. Radiological assessment *in vivo* is associated with limited reliability, particularly in accurately predicting implant loosening due to inadequate osseointegration. This may be related to the limited spatial resolution of current imaging equipment and due to metal artefacts that obscure the interface between implant and bone [9].

Photon-counting detector (PCD) CT is a novel technology that might offer a solution for adequate *in vivo* assessment of osseointegration. The improved detector technology offers superior spatial resolution, lower noise and possibly fewer metal artefacts than conventional, energy-integrating detector CT (EID-CT) [10]. In EID, x-rays are converted to an electric signal using a two-step process. First, x-rays are converted to visible light using a scintillator. Then, this light is converted to an electric signal. In this process the energy of the x-ray particles cannot be measured. In PCD-CT, x-rays are directly converted to an electric signal. Due to a simplified detector geometry containing a single semiconductor layer, geometrical dose efficiency is improved. Also, pixels can be made smaller allowing for ultra-high-resolution imaging. Finally, due to the ability to measure the energy of individual x-ray photons, electronic noise can be effectively removed, enabling either a higher contrast-to-noise ratio or reduced radiation dose. Compared to the EID-CT, the PCD-CT has been shown to improve the visualization of anatomic detail in musculoskeletal applications, including spine [11], wrist [12–15], shoulder, and pelvis [16].

The primary aim of this work was to assess the image quality for assessment of osseointegration (bone-implant interface, trabecular structure, and image noise) comparing PCD-CT with conventional EID-CT. The secondary aim was to quantitatively measure the sharpness of the bone-implant interface in the images acquired using PCD-CT and EID-CT. We hypothesized that image quality for assessment of the bone-implant interface and osseointegration would be superior in PCD-CT compared to EID-CT.

## Methods

### Implants and image acquisition

**Fig. 2:**
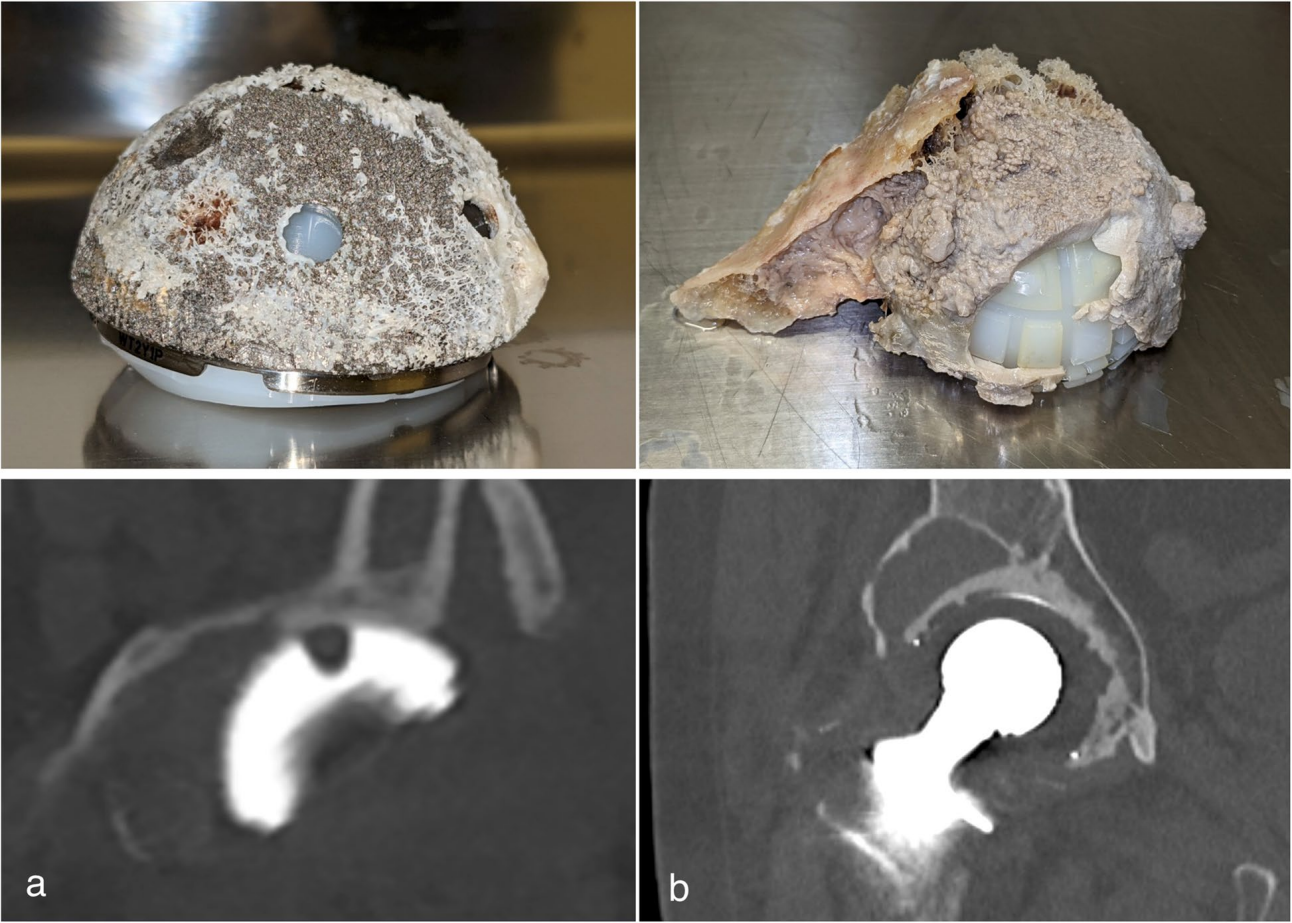
Retrieved uncemented (**a**) and cemented (**b**) acetabular cup implant and clinical computed tomography images acquired before revision surgery (lower row)

Two acetabular implants, one cemented (Lubinus Acetabular Cup, Waldemar Link GmbH & Co. KG) and one uncemented (Trident® Tritanium™, revision, Acetabular System, Stryker Orthopaedics), retrieved during revision surgery (Fig. 2) were scanned with an optimized high-resolution bone protocol on a clinical PCD-CT scanner (NAEOTOM Alpha, Siemens Healthineers with Syngo CT version VA40) and an EID-CT (SOMATOM Force, Siemens Healthineers with Syngo CT VB20) with matching CTDIvol value (12.2 mGy), pitch (0.5), and rotation time (1.0 s).

For PCD-CT, collimation was 120 × 0.2 mm, and for EID-CT, collimation was 64 × 0.6 mm (*z*-sampling), representing the narrowest collimation possible on each scanner. For optimal reduction of metal artefacts, both scanners used an additional tin (Sn) filtration applied to the x-ray tube, which operated at 140 kVp for PCD-CT and 150 kVp for EID-CT. Tube current modulation was turned off for all scans. The study was approved by the Swedish Ethical Review Authority (Dnr 2022–05,803-02).

### Image reconstruction and post-processing

For EID-CT, images were reconstructed using a clinically used reconstruction kernel (Ur77) and additionally at a sharper kernel strength (Ur89) to evaluate the maximum spatial resolution possible on the EID-CT. For PCD-CT, images were reconstructed using kernels optimized for bone, that matched the kernels used with EID-CT in terms of sharpness (modulation transfer function, MTF): Br76 and Br89. Additional reconstructions were made for PCD-CT using sharper kernels (Br92 and Br98) to evaluate the maximum spatial resolution possible on the PCD-CT. Matrix size was 512 × 512 for reconstructions with Ur77 and Br76 kernels, and 1024 × 1024 for reconstructions with Br89, Br92, and Br98 kernels. Field-of-view was 50 mm for all reconstructions. Slice thickness and increments were 0.4/0.1 mm for EID-CT and 0.2/0.1 mm for PCD-CT, *i.e.*, the best possible setting for each scanner. For EID-CT images, iterative reconstruction strength 4 (80%) of the Advanced Modeled Iterative REconstruction (ADMIRE) technique was used. For PCD-CT images, Quantum Iterative Reconstruction (QIR) strength 3 (75%) and 4 (100%) were used. QIR strength is specified at the end of the kernel name, *i.e.*, Br92\4 indicates the Br92 kernel with QIR strength 4.

### Subjective image quality assessment

Four radiologists reviewed the image stacks in a side-by-side fashion, blinded to the CT scanner and reconstruction parameters. A total of 10 image stacks were presented using different combinations of kernel strength and iterative strength (EID-CT: Ur77\4, Ur89\4; PCD-CT: Br76\3, Br76\4, Br89\3, Br89\4, Br92\3, Br92\4, Br98\3, Br98\4). Images were anonymized and displayed in random order on a clinical workstation. Observers were allowed to pan and zoom images freely and to change the window width and center, which were initially set at a bone setting (3,000/500 Hounsfield units). Criteria and rating scale used for assessing image quality in images of cemented and uncemented cups are presented in Table 1.

**Table 1:** Image quality criteria and rating scale used for the subjective image quality assessment

Criteria
Cemented cup
1. There is a sharp delineation of the bone/cement interface
2. There is a sharp delineation of cement/poly interface
3. The image noise does not interfere with my clinical assessment
4. There is a sharp delineation of the trabecular bone structure
Uncemented cup
1. There is a sharp delineation of the bone/implant interface
2. There is a sharp delineation of titanium/poly interface
3. The image noise does not interfere with my clinical assessment
4. There is a sharp delineation of the trabecular bone structure
Rating scale
1. I am sure that the criterion is not fulfilled
2. I am almost sure that the criterion is not fulfilled
3. I am not sure if the criterion is fulfilled or not
4. I am almost sure that the criterion is fulfilled
5. I am sure that the criterion is fulfilled

### Quantitative measurements

Analysis of the image stacks was done using Fiji v2.3.0/1.53f [17]. The sharpness of the bone-cement interface in the cemented cup and the bone-titanium interface in the uncemented cup was measured by fitting CT numbers along a line across the interface to a mathematical error function, which is defined as

$$\mathrm{y}=b+c\bullet erf\left(\frac{x}{d}\right)$$

where *b* is the CT number in the bone, *c* is the difference in CT numbers across the interface, and *d* is the slope. From *d*, the full width at half maximum (FWHM) of the underlying Gaussian function can be derived:

$$FWHM = d\bullet 2\sqrt{\mathrm{ln}2}$$

**Fig. 3:**
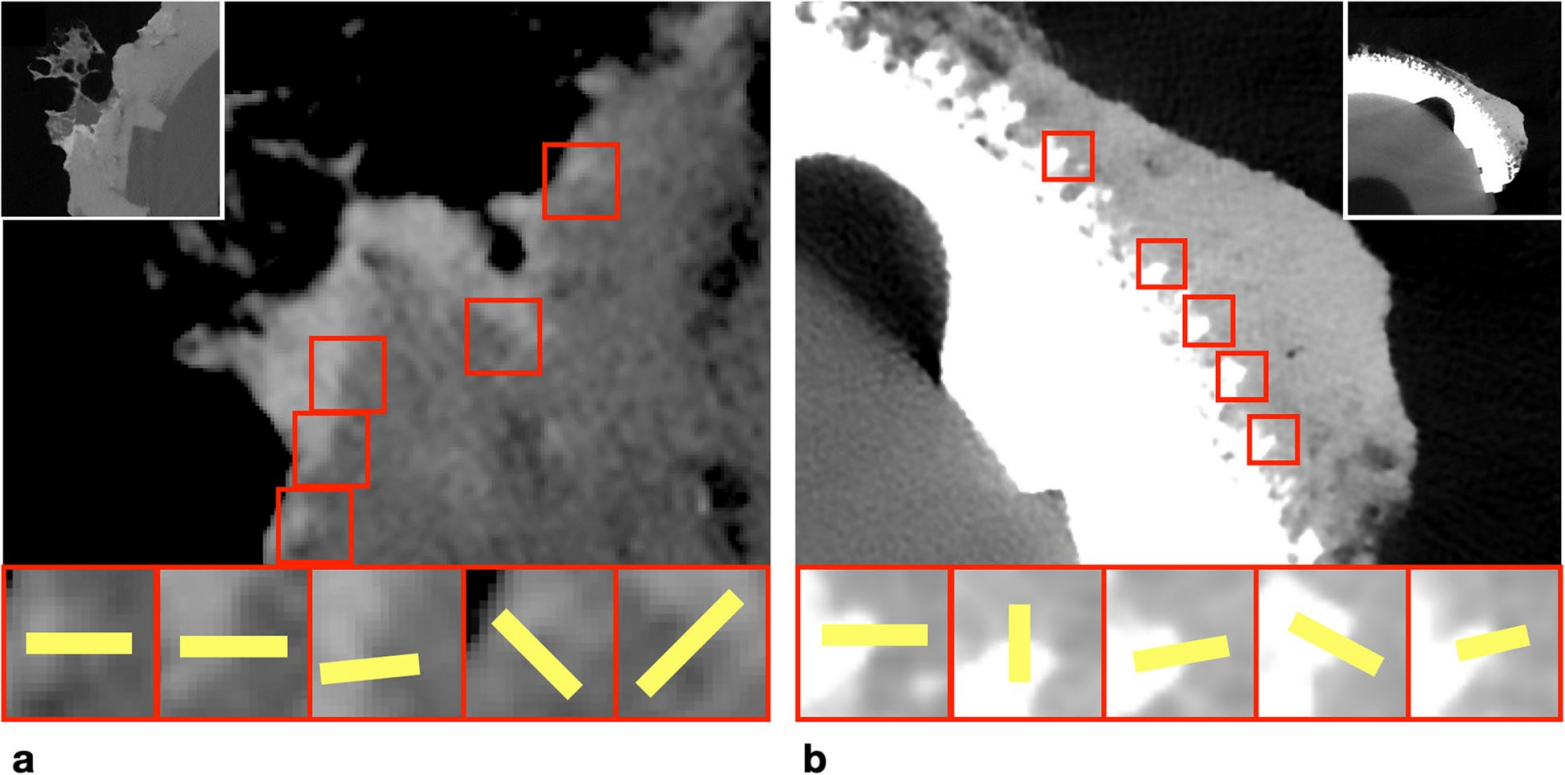
Positions of the interface sharpness measurements in the cemented cup (**a**) and the uncemented cup (**b**). Both images were obtained with photon-counting detector computed tomography, using reconstruction kernel Br98 with quantum iterative strength 4

The FWHM is thus a measure of sharpness of the interface, with a smaller value indicating a sharper edge. The FWHM was measured at 5 fixed locations for each type of cup (Fig. 3).

### Data analysis

Image quality ratings were expressed as median and interquartile range (IQR) and were analyzed using Wilcoxon matched pairs signed rank tests to test for differences between scanners, kernels, and iterative strengths. Interface sharpness (FWHM) was measured at 5 different positions for each interface (bone-cement and bone-titanium) and was expressed as mean and standard deviation. Differences in FWHM were analyzed using one-way repeated measures analysis of variance with Dunnet’s correction for multiple comparisons. All analyses were done using GraphPad Prism version 9.0, (GraphPad Software). A *p* value below 0.05 was considered statistically significant.

## Results

### Subjective image quality assessment

**Fig. 4:**
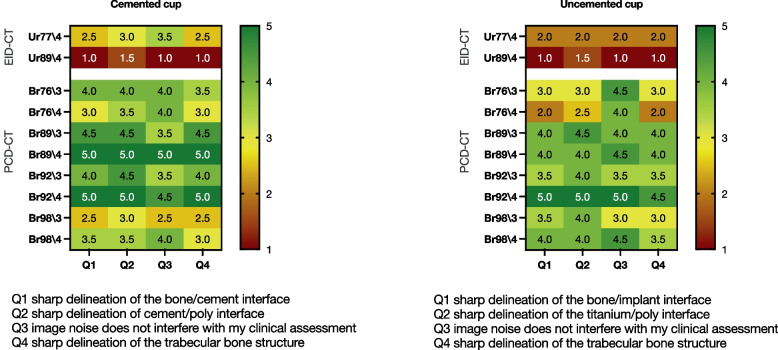
Median reader scores for 4 different criteria (Q1–Q4) for acetabular cup images obtained using energy-integrating detector computed tomography (EID-CT, top 2 rows) and photon-counting detector computed tomography (PCD-CT, bottom 8 rows) with different reconstruction kernels. Left: cemented cup; Right: uncemented cup

Figure 4 shows the median ratings given by the four readers for each criterion and each combination of kernel and iterative strength, for cemented and uncemented cups. The median Likert scores across all criteria were higher for PCD-CT than EID-CT at comparable kernel strength, but only when QIR3 was used, both in the cemented cup (Ur77\4: median 3, IQR 2.00–3.75; Br76\3: median 4, IQR 3.00–4.75, *p* = 0.02) and in the uncemented cup (Ur77\4: median 2, IQR 2.00–2.00; Br76\3: median 3. IQR 3.00–4.00, *p* < 0.001). Reader scores further increased with increasing kernel strength, but only for PCD-CT.

**Fig. 5:**
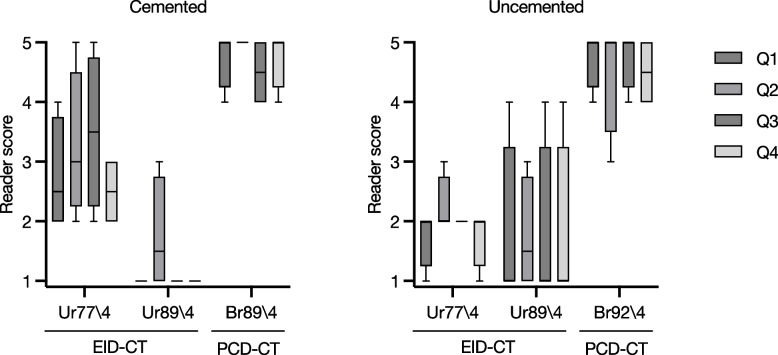
Reader scores for 4 different criteria (Q1–Q4) for photon-counting detector computed tomography (PCD-CT, reconstruction kernels Br89\4 and Br92\4), compared to energy-integrating detector computed tomography (EID-CT, Ur77\4). Boxes indicate interquartile range; whiskers indicate minimum and maximum values

Br89\4 was considered to result in the best image quality across all four criteria for the cemented cup (median 5, IQR 4.25–5.00), while Br92\4 yielded the highest reader scores for the uncemented cup (median 5.00, IQR 4.00–5.00). To compare the best results for PCD-CT (Br92\4 and Br89\4) with the best results for EID-CT (Ur77\4) we compared the reader scores in Fig. 5. Scores were higher with both PCD-CT kernels and for all criteria (Q1, *p* = 0.016; Q2, *p* = 0.023; Q3, *p* = 0.031; Q4, *p* = 0.008).

### Quantification of bone-implant and bone-cement interface sharpness

**Fig. 6:**
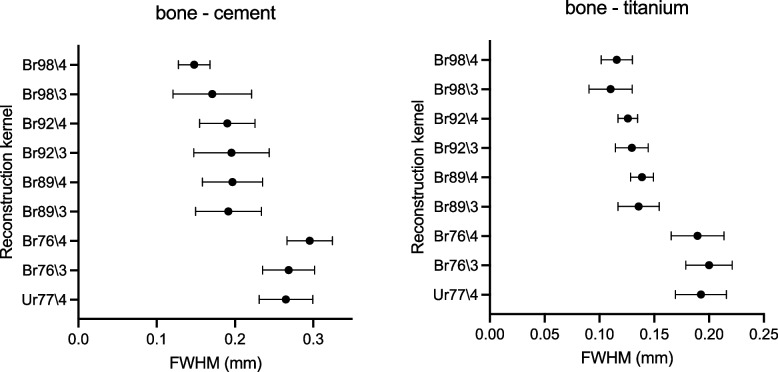
Sharpness of the interface between bone and cement (left) in the cemented cup, and between bone and titanium (right) in the uncemented cup, measured as full-width half-maximum (FWHM) value (mm), for energy-integrating detector computed tomography (EID-CT) and photon-counting detector computed tomography (PCD-CT) using different reconstruction kernels

Figure 6 shows the results of the quantitative measurements of the interface sharpness. In the cemented cup, the sharpness of the bone-cement interface was similar in PCD-CT images compared to EID-CT images, when reconstructed using comparable kernels (Br76\3, FWHM 0.27 ± 0.03 mm [mean ± standard deviation] *versus* Ur77\4, 0.27 ± 0.03 mm). Sharpness was increased with PCD-CT at increasing kernel strength, with the lowest FWHM of 0.15 ± 0.02 mm at Br98\4 (*p* < 0.001). In the uncemented cup, the sharpness of the bone-titanium interface was similar between EID-CT and PCD-CT for comparable kernel strength (Br76\3, FWHM 0.20 ± 0.02 mm *versus* Ur77\4, 0.19 ± 0.02 mm) and FWHM decreased with increasing kernel strength. The lowest FWHM was 0.11 ± 0.02 mm using Br98\3, *i.e.*, significantly better sharpness than with EID-CT, Ur77\4, *p* < 0.001). Iterative strength in PCD-CT reconstructions did not affect the interface sharpness significantly. For EID-CT with kernel Ur89, the FWHM could not be determined due to excessive noise in the images.

## Discussion

**Fig. 7:**
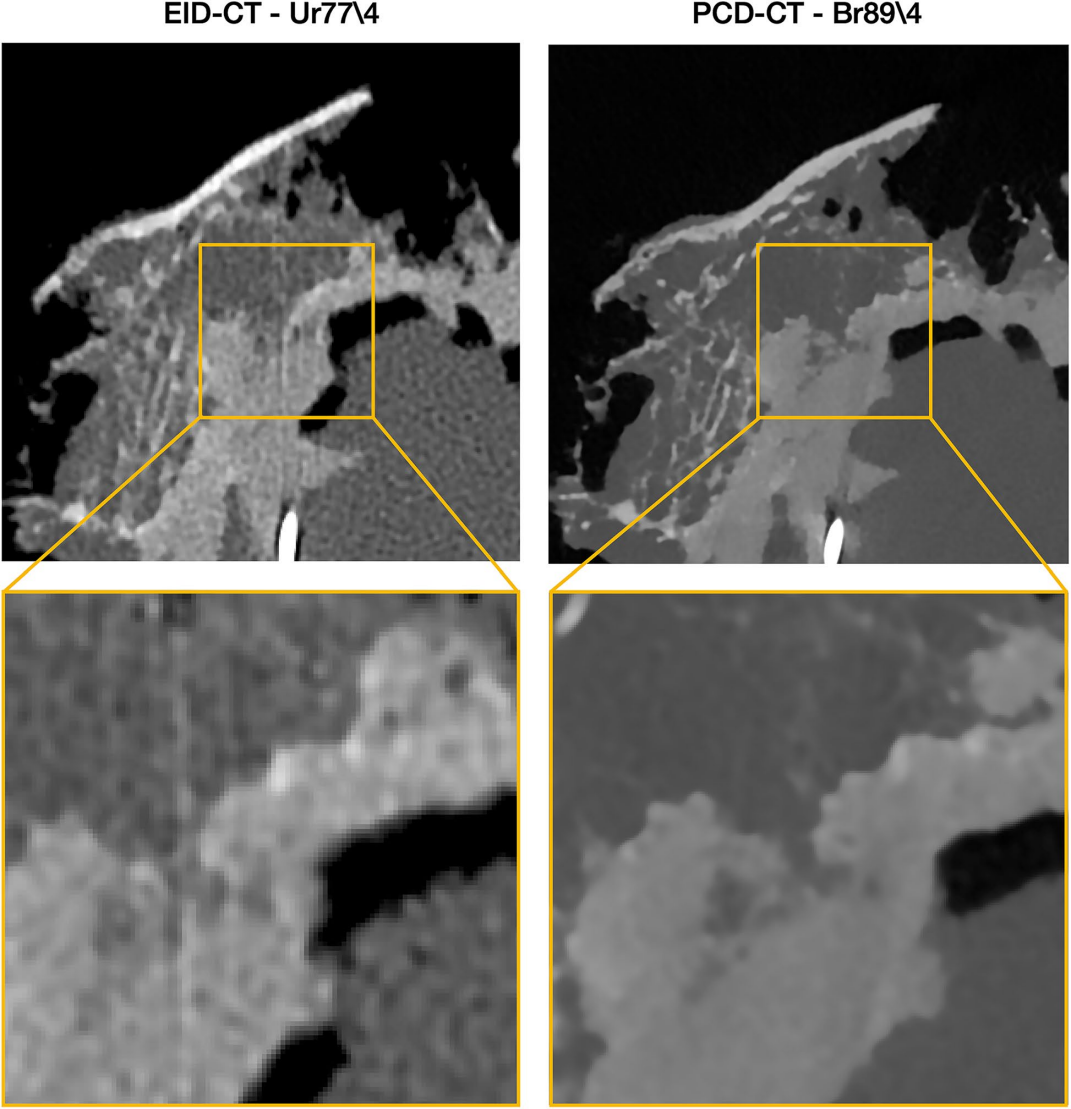
Example of images obtained with EID-CT using Ur77 reconstruction kernel with iterative strength (ADMIRE) 4 and 0.4 mm slice thickness (left) and photon-counting detector computed tomography (PCD-CT) using a Br89 kernel with quantum iterative strength (QIR) 4 and 0.2-mm collimation (right). Differences in visualization quality of the cement–bone interface in the cemented cup can be clearly observed

We hypothesized that PCD-CT would improve the assessment of the bone implant interface and osseointegration in cemented and uncemented acetabular cups when compared to EID-CT. In our comparisons, we found superior ratings of all aspects of image quality by radiologist, both for the cemented and the uncemented implant (Figs. 4 and 7). The superior ratings were supported by objective improvements in sharpness of the bone-implant interface.

Clinicians rated the PCD-CT images, reconstructed with the sharp Br89 kernel, at a median score of 5 for the cemented cup and with the Br92/4 kernel in both the cemented and uncemented cup. The scores indicate that the delineation of the interface between bone and implant was considered sharp. Compared with EID-CT, the best rating for evaluation of the bone-implant interface was 2.5 for the cemented cup and 2.0 for the uncemented cup with the best kernel (Ur77/4). Improvements in the radiologist’s visual assessment of image quality were robust and independent of the specific type of image assessment or type of fixation (cemented or uncemented). Even reconstruction kernels with the lowest visual assessment results in PCD-CT were comparable with results from the best EID-CT kernel. There was a slight difference in the perceived optimal kernel strength of PCD-CT for the cemented cup (Br89\4) compared to the uncemented cup (Br92\4) and it appeared that the uncemented cup was in general more difficult to evaluate regarding all aspects of our visual assessment. These differences might be related to the material properties of the titanium and related difficulties to assess the interface due to metal artefacts. Another explanation might be the differences between the two specimens. The cemented cup had a larger piece of trabecular bone structure attached to the cup, while the uncemented cup showed a cortical bone structure with a much smaller volume (Fig. 2). An increase in kernel strength for the EID (Ur89\4) instead of the clinical standard at our unit, Ur77\4, resulted in deteriorations in the assessment of the image due to excessive image noise. The optimal iterative strength in assessment of image quality was the ADMIRE 4 for EID-CT and QIR4 for PCD-CT. It appears that clinicians preferred a stronger noise reduction, and that this reduction was not associated with any negative effects on the visualization of the bone-implant interface.

Direct assessment of osseointegration *in vivo* remains a diagnostic challenge despite the availability of advanced imaging modalities such as Fluorine-18-fluorodeoxyglucose positron emission tomography, single-photon emission computed tomography/computed tomography, dual-energy x-ray absorptiometry, magnetic resonance imaging, as well as subtraction arthrography, nuclear arthrography and bone scintigraphy [18–24]. None of the methods has gained wider popularity in clinical use due to limitations in clinical feasibility, cost, and diagnostic accuracy [24].

Because evaluation of osseointegration *in vivo* is not available in clinical practice, adequate osseointegration is typically inferred from the absence of certain radiological findings, such as radiolucent lines. In cemented cups, assessment of osseointegration/implant fixation includes the occurrence of radiolucent lines on radiographs, and their thickness and extension around the circumference of the cup [25]. The thicker the radiolucent line and the larger the demarcation, the higher the likelihood for the implant to be found loose during revision surgery. However, the predictive value of these signs is not high enough to reliably assume adequate osseointegration in the absence of these signs. Techniques involving the assessment of implant migration over time, radiostereometric analysis [26], and Einzel-Bild-Roentgen-analysis [27] have a good predictive value for aseptic loosening, but are dependent on the analysis of sequential radiographs with specific protocols, making it a popular tool in research but unfeasible for clinical use.

Our results are encouraging because PCD-CT offers improved spatial resolution, lower noise and potentially even better metal artefact reduction than EID-CT [11, 12]. All of these features have the potential to fill the technological gap of *in vivo* diagnostics of osseointegration in orthopedic implants. The advantages of PCD-CT are the smaller detector elements compared to EID-CT, which substantially improves the spatial resolution in the PCD-CT images [10]. Due to the direct conversion from x-rays to electrical signals and the relatively strong weighing of lower-energy photons, the geometric dose efficiency is increased as well [28]. The resulting improvement in image sharpness together with the lower noise levels makes it possible to use sharper reconstruction kernels highlighting smaller details in the images while maintaining acceptable image noise levels. The number of studies investigating the visibility of the interface between bone and implant material is limited. Lau et al. [29] found that polyethylene insert wear and metallic tibial tray wear could be detected by multi-energy PCD-CT. Other studies have shown improved visibility of bone structures in the wrist [13, 30], the shoulder and the pelvis [16]. The PCD-CT system in our study uses additional tin filtration and an extended Hounsfield scale, reducing the magnitude of metal artefacts [31]. This artefact reduction may also have been beneficial in the visualization of the bone-metal interface in our study.

We used high tube voltages to reduce beam hardening and metal artefacts [32], and because higher energy photons are more likely to reach the detector even in the presence of metal objects. More photons at the detector lead to improved image quality and therefore, at least theoretically, improved diagnostic accuracy. These considerations are in line with the “as low as reasonably achievable”, ALARA, principle, even if radiation dose might be increased. A formal evaluation of tube voltage and filtration on radiation dose and image quality was beyond the scope of this study.

Our study has several limitations. We investigated only two different extracted joint implants, representing only a small sample of a large variety of polyethylene and titanium alloys currently used in joint replacement products. Also, the contact area of bone with the implant was very limited compared to the *in vivo* situation and the amount of bone and type of bone tissue remnants differed between the cemented and uncemented implant. Finally, the study was carried out using the first software version of the commercially available PCD-CT, NAEOTOM Alpha (Siemens Healthineers). As the system’s software is continuously updated, newer versions might yield different results.

In conclusion, this study shows improved visualization of bone-implant interface in *ex vivo* samples of acetabular cups using PCD-CT compared to EID-CT, suggesting a potential use of PCD-CT for direct evaluation of osseointegration. Prospective studies in patients are required to assess image quality of PCD-CT *in vivo* and to further investigate its clinical value for the assessment of osseointegration after joint replacement surgery.

## Data Availability

The datasets used and/or analyzed during the current study are available from the corresponding author on reasonable request.

## Abbreviations

CT: Computed tomography
EID-CT: Energy-integrated detector computed tomography
FWHM: Full width half maximum
PCD-CT: Photon-counting detector computed tomography
QIR: Quantum iterative reconstruction
TJR: Total joint replacements
